# An individual with multiple high-risk links uncovered: an epidemiological investigation based on molecular network analysis

**DOI:** 10.3389/fpubh.2026.1715356

**Published:** 2026-02-04

**Authors:** Shijiao He, ShanShan Li, Qinying He, Fang Liu, Zhen Dai, Minjing Li, Fanghong Gong, Zhenhua Duan, Liang Wang

**Affiliations:** 1 Department of Sexually Transmitted Diseases and HIV Prevention and Control, Chengdu Center for Disease Control and Prevention (Chengdu Institute of Health Supervision), Chengdu, Sichuan, China; 2 Department of HIV Testing, Chengdu Center for Disease Control and Prevention (Chengdu Institute of Health Supervision), Chengdu, Sichuan, China

**Keywords:** epidemiological investigation, HIV-1, men who have sex with men, molecular cluster, phylogenetic analysis

## Abstract

**Background:**

In December 2023, the local HIV confirmatory laboratory in a Southwestern city of China conducted molecular network analysis on newly reported cases of young students and nonstudent men who have sex with men (MSM) from 2018 to 2023 and identified a key molecular cluster. The aim of the study is to analyze the transmission network characteristics and elucidate the potential transmission dynamics within this cluster, and prevent further spread of the disease.

**Methods:**

A cluster was designated as a key molecular cluster based on node characteristics, internal network density, and whether young students were involved. For the key cluster, a face-to-face epidemiological investigation was conducted, and detailed information on sociodemographic characteristics, histories of high-risk sexual behavior and sexual partners was collected. For those individuals identified through sexual partner tracing, HIV nucleic acid extraction, HIV-1 *pol* gene amplification, and sequencing were performed. Molecular network and ML phylogenetic tree analyses were conducted to verify whether they belong to the same molecular cluster.

**Results:**

All 20 individuals in the key molecular cluster were male, with 9 being young students and 11 being nonstudent MSM The investigations revealed that three young students (A, B, C) provided the geosocial networking app (Blued) account information of the same high-risk sexual partner (H, was not included in the aforementioned molecular cluster), who actively solicited unprotected anal sex with these three individuals. H was contacted through Blued by local MSM organizations, and tested HIV-positive. After sexual partner tracing of H, he provided information on one high-risk sexual partner (D). Cross-referencing H’s Blued data with local AIDS sexual partner tracing databases linked him to two young students diagnosed with HIV (E, F). Laboratory molecular network analysis confirmed that H belonged to the same molecular cluster as the aforementioned six people living with HIV (PLWH).

**Conclusion:**

By combining molecular transmission network analysis with epidemiological investigations, one individual with multiple high-risk sexual links who was initially not included in the molecular cluster was promptly detected, potentially preventing further transmission by creating awareness of HIV status and provision of HIV treatment services. It is necessary to improve the molecular network monitoring and conduct high-quality epidemiological investigation on the identified key molecular clusters as soon as possible to find persons who would benefit from HIV treatment and prevention services.

## 1 Introduction

The HIV/AIDS pandemic continues to pose a formidable challenge to global health. According to the latest estimates from the Joint United Nations Programme on HIV/AIDS (UNAIDS), approximately 40.8 million people were living with HIV globally in 2024, indicating a significant disease burden (1). Despite significant advancements in prevention, treatment, and care, the epidemic remains a major public health concern, as evidenced by an annual incidence of 1.3 million new infections and a staggering 630,000 AIDS-related deaths (1). The burden of disease is particularly pronounced in low- and middle-income countries, where access to healthcare resources is often limited, highlighting the critical importance of targeted epidemiological surveillance and interventions (2).

A molecular cluster refers to a group of individuals diagnosed with HIV infection whose viral strains exhibit a high degree of genetic sequence similarity as determined through phylogenetic analysis. This assessment of similarity depends on specific genetic distance thresholds and the selection of a background sequence dataset. Due to the constant evolution of HIV, highly similar sequences may suggest potential epidemiological links between cases. However, molecular data alone cannot confirm direct transmission relationships or infer the direction of transmission. Sequence similarity may result from indirect transmission chains or unsampled intermediate cases; therefore, a molecular cluster only indicates potential transmission links. Once a molecular cluster is identified, the corresponding transmission cluster and risk networks can be identified only through investigation (3). Traditional HIV epidemiological investigations and partner tracing often involve biases due to the sensitive nature of the information collected. Molecular networks can effectively supplement traditional epidemiological and social network data, accurately identify transmission clusters, and further assess the results of social network analysis in epidemiological investigations (4–6).

Molecular networks were used to supplement the HIV transmission relationships that could not be identified by social network analysis. The integration of epidemiological surveys with molecular network analysis has emerged as a powerful tool in the fight against HIV/AIDS. This synergy between epidemiological and molecular methods not only enhances our understanding of epidemic dynamics but also facilitates early case detection and timely interventions, as evidenced by the successful application of this approach in various regions (7–9). HIV phylogenies based on sequence similarity and inference of common ancestors can identify putative transmission clusters (10, 11). Rapidly detecting and responding to emerging clusters of HIV infection to further reduce new transmission has been listed as one of the four strategic initiatives, including a strategic initiative by the US Department of Health and Human Service, which has good application prospects in the field of AIDS prevention and treatment (12).

In China, a network centered on CDCs (Centers for Disease Control and Prevention) and covering medical institutions at all levels as well as community-based organizations has been built. Leveraging strategies such as Provider-Initiated Testing and Counseling (PITC), expanded testing among key populations, and self-testing, the early detection rate of HIV infections has been enhanced (13). For PLWH identified through testing, partner notification serves as a critical link in interrupting transmission. To further improve the accuracy of detection and identification, China’s Plan for Combating and Preventing AIDS (2024–2030) explicitly requires the use of viral gene sequencing and molecular networks to conduct epidemiological investigations of newly reported cases (14).

Amidst the global HIV/AIDS landscape, the epidemic among young students has emerged as a particularly pressing concern. In China, where the overall HIV epidemic has shown signs of stabilization, considering the particularity of young students and their important impact on future society, it is one of the key populations for HIV prevention and control (15). In recent years, the number of newly reported HIV/AIDS cases in a western city of China has shown a downward trend, but this trend has not been observed among young students. To explore the potential sources of infection and analyze the possible transmission dynamics in this population, the city launched molecular network surveillance of newly reported HIV/AIDS cases in young students in 2021. Owing to the relatively small sample size of young students, it was difficult to form clusters in separate analyses. Considering that young students are infected mainly by homosexual sex, the gene sequences of newly reported nonstudent MSM cases were included in the analysis beginning in 2022.

In December 2023, the local HIV confirmatory laboratory conducted molecular network analysis on newly reported cases of young students and nonstudent MSM from 2018 to 2023 and identified a key molecular cluster. To assess potential molecular association, identify potential infections in the transmission chain, and prevent further spread of the disease, epidemiological investigations were carried out immediately.

## 2 Methods

### 2.1 Subjects for molecular network surveillance

Newly diagnosed HIV-positive individuals were flagged through the National HIV/AIDS Surveillance Information System—a nationwide unified public health platform managed by the Chinese Center for Disease Control and Prevention (China CDC) in accordance with the Infectious Diseases Prevention and Control Law and Regulations on the Prevention and Control of AIDS. Molecular network surveillance was conducted on PLWH diagnosed in a western city of China from 2018 to 2023. HIV-1 *pol* gene sequencing was performed using pre-antiretroviral therapy plasma samples provided by HIV testing laboratories.

### 2.2 Sequencing of the HIV-1 pol gene

HIV nucleic acid extraction was performed via the Xi’an Tianlong Automatic Nucleic Acid Extraction System (GeneRotex96). RT-PCR and nested PCR were employed to amplify the HIV-1 *pol* gene protease (full length) and the reverse transcriptase region (the first 300 amino acid sites), with an amplification product size of 1,316 bp. The PCR conditions and amplification primers were executed according to the research in Sichuan province (16). The first round of RT-PCR and the second round of PCR were conducted via the Promega (A1702) Kit from the USA and the TIANGEN (KP201) Kit, respectively. PCR products were observed by 1% agarose gel electrophoresis, and amplification products with bands appearing at 1200 bp were sent to Beijing Nuosai Genomics Research Center Co., Ltd. or Beijing Anpu Biochemical Technology Co., Ltd., for purification and gene sequencing.

### 2.3 Sequence quality control and processing

To ensure the sequences used for molecular network analysis were of high quality and free from contamination, we implemented a rigorous three-stage quality control (QC) workflow: (1) Sequencing Stage: ensuring clear chromatograms, >95% bidirectional sequence coverage, and no significant mixed peaks; (2) Assembly and Alignment Stage: using Sequencher 5.0 software, requiring the assembled consensus sequence length to be >1,000 bp, the proportion of ambiguous bases to be <5%, and correct alignment with reference sequences; (3) Pre-submission Stage: performing manual inspection for obvious signs of PCR recombination or contamination, and removing any low-quality sequences. Laboratory testing and data analysis quality control were conducted in accordance with the Guidelines for HIV Transmission Network Surveillance and Intervention of China (17).

### 2.4 Molecular network construction

The ClustalW method in BioEdit 7.2.0 software was used to align and correct the prepared sequences with international reference strains (from the HIV sequence database of the Los Alamos National Laboratory in the USA). The genetic subtype of each sequence was determined by constructing a maximum likelihood (ML) phylogenetic tree using FastTree. TN93 nucleotide substitution model implemented in Hyphy 2.2.4 was used to calculate pairwise genetic distances between sequences. This threshold range (0.005 to 0.015) was chosen on the basis of previous studies of the HIV-1 *pol* gene region and is generally effective in identifying recent transmission events (10, 18). We tested multiple thresholds across this range and selected a single threshold that yielded the largest number of propagation clusters for the final network construction and cluster identification. The HIV molecular transmission network diagram is constructed using the Cytoscape 3.6.1 software.

A cluster was identified as a key molecular cluster if it satisfied all of the following criteria:

1. Consisting of at least 10 nodes (defined as 10 PLWH with valid sequence data);
2. Displaying a higher internal network density (defined as the ratio of actual genetic edges [pairwise distances ≤ threshold] to the total possible edges in the cluster, which exceeded the mean density of all other clusters with ≥10 nodes) compared to other clusters;
3. Encompassing a relatively high proportion of cases involving young students (defined as≥30% of cluster members being young students).

### 2.5 Epidemiological investigation

The epidemiological investigation was conducted from December 1, 2023, to July 1, 2024, in a western city of China. All PLWH within the molecular transmission clusters underwent epidemiological investigations conducted by local CDC professionals.

The investigations were carried out in AIDS Voluntary Counseling and Testing (VCT) clinics with good privacy protection. All participants have signed informed consent forms. The survey content included demographic information (gender, age, education level), high-risk behavior characteristics (refer to self-reported behaviors prior to diagnosis that are epidemiologically established to significantly increase the risk of acquiring or transmitting HIV), core information of high-risk sexual partners (name, nickname, contact phone number, geosocial networking app account), and HIV testing history.

In this study, to better understand potential transmission networks and support epidemiological investigations, we collected information on participants’ use of social applications, including Blued—a widely used mobile app among global and Chinese MSM communities. Blued facilitates location-based social matching, real-time communication, community updates, and health information access. Within China, it serves as a common platform for online socializing, connection-building, and organizing offline activities, making it a relevant digital trace in HIV transmission network investigations. Data regarding app usage were obtained solely through epidemiological interviews.

### 2.6 High-risk contact tracing

In this survey, the tracing of high-risk sexual partners mainly relied on local social organizations serving MSM. These organizations usually carry out long-term activities for the MSM community in local areas, such as health education and HIV testing promotion. Owing to their characteristic of being deeply embedded in the community, such organizations often establish cooperation mechanisms with local public health authorities. With the informed consent of people living with HIV, these organizations can leverage their community networks and trusted relationships to mobilize high-risk contacts to undergo testing. All information related to the identities of participants is strictly managed by public health institutions. When assisting with outreach, community organizations only deliver invitations to participate in the study, and shall not disclose any content related to the HIV status or personal information of people living with HIV.

Since 2021, this region has established a database for source tracing investigations of newly reported PLWH. This is an operational database formed by public health institutions in the course of AIDS case management and sexual partner tracing, in accordance with regulations such as the Regulation on the Prevention and Treatment of HIV/AIDS. In this study, the contact information obtained through field epidemiological investigations will be cross-validated and compared with the records in this database to supplement and verify clues related to the chain of transmission.

### 2.7 Molecular analysis of transmission links

Plasma samples were immediately collected before antiretroviral therapy for sequencing after confirming the infection of key high-risk sexual partners identified through field epidemiological investigations, and molecular network analysis is then conducted to determine the existence of molecular associations and whether they belonged to the same molecular cluster. In addition, a ML phylogenetic tree was constructed using MEGA 7.0 to assess sequence homology. The tree was inferred under the GTR + G + I model with 1,000 bootstrap replicates and visualized using FigTree 1.4.5. To evaluate the homology between the target sequences and their position in the local genetic background, we screened 30 background sequences from our laboratory database. The screening criteria were as follows: (1) from the same city (2018–2023); (2) the transmission route was MSM; (3) aged between 18 and 30 years old; (4) qualified sequence quality (length >1,000 bp, mixed base <5%); (5) high genetic similarity to the target sequence (preliminary distance analysis) to ensure that the phylogenetic tree is locally representative.

## 3 Results

### 3.1 Basic characteristics of HIV/AIDS cases within the molecular cluster

**Figure 1:**
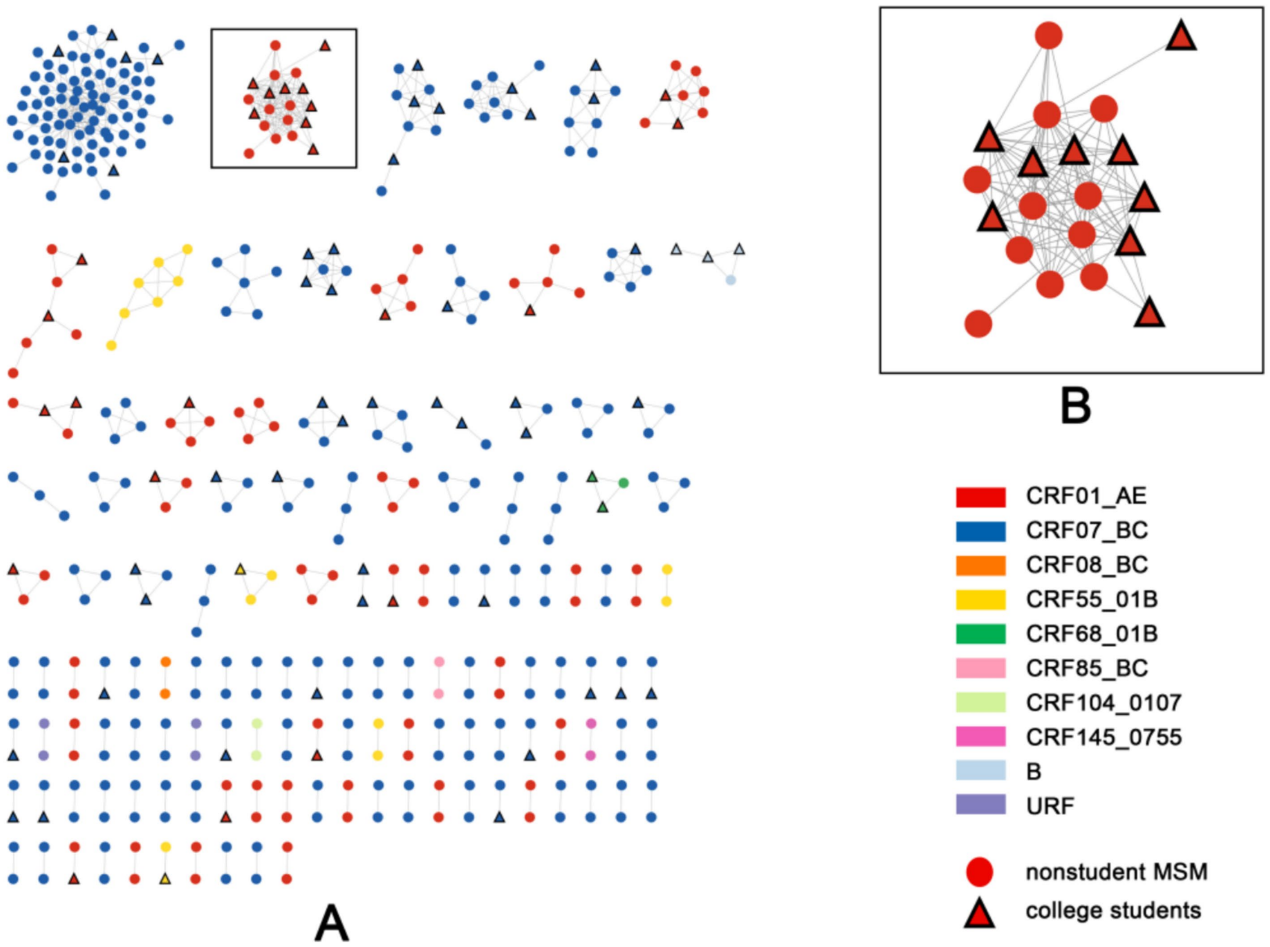
Molecular transmission network among young students and nonstudent MSM in a western Chinese city. **(A)** Overall transmission network: Constructed using a genetic distance threshold of 0.007, where nodes represent PIWH (triangles = young students; circles = nonstudent MSM), and colors indicate HIV-1 subtypes (CRF01_AE, CRF07_BC, etc.) and unassigned recombinant forms (URF). **(B)** Identified key transmission cluster: This cluster consists of 20 nodes (9 young students, 11 nonstudent MSM) and exhibits the highest internal density (0.58) among clusters with ≥10 nodes.

A key cluster was identified within the molecular surveillance networks of young students and non-student MSM diagnosed between 2018 and 2023 (Figure 1). This cluster belonged to the CRF01_AE subtype, comprised 20 nodes, and exhibited the highest network density (0.58) among all large clusters (≥10 nodes). Young students accounted for 45% (9/20) of the individuals within this cluster.

The 20 PLWH were all males, with 9 being students and 11 being nonstudent MSM. The youngest patient was 17 years old, and the oldest patient was 31 years old, with an average age of 21.05 ± 2.89 years. Three cases were confirmed in 2021, eight in 2022, and nine in 2023.

### 3.2 Epidemiological investigation of HIV/AIDS cases within the molecular cluster

Except for 5 PLWH who are not currently residing in the local area, epidemiological investigations were completed for the remaining 15 cases. The investigations revealed that three young students (A: confirmed on January 8, 2023, with acute-phase infection; B: confirmed on April 17, 2023; C: confirmed on August 19, 2022) provided the Blued account information of the same high-risk sexual partner (H).

**Figure 2:**
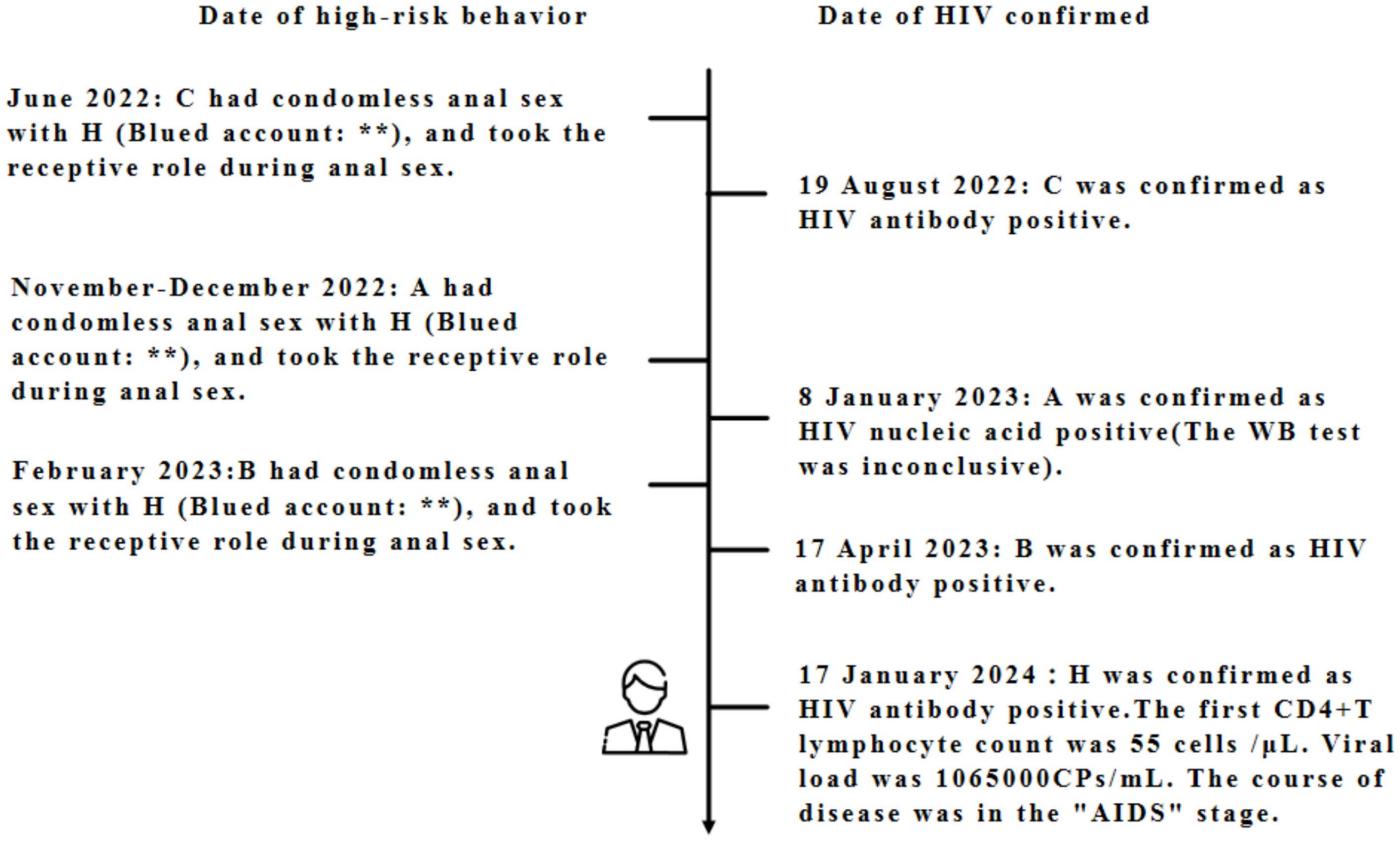
Timeline of high-risk sexual encounters and HIV diagnosis among (A–C, H). The timeline is not to scale. This timeline depicts self-reported high-risk sexual encounters and HIV diagnosis dates; it does not confirm direct transmission between individuals, only potential epidemiological links.

H actively solicited unprotected anal sex with these three young students through Blued in November–December 2022, February 2023, and June 2022, respectively. The local social organizations serving the MSM community contacted H through Blued, which confirmed his infection on January 17, 2024 (with an initial CD4^+^ T lymphocyte count of 55 cells/μL and a viral load of 1,065,000 CPs/mL) (Figure 2).

**Figure 3:**
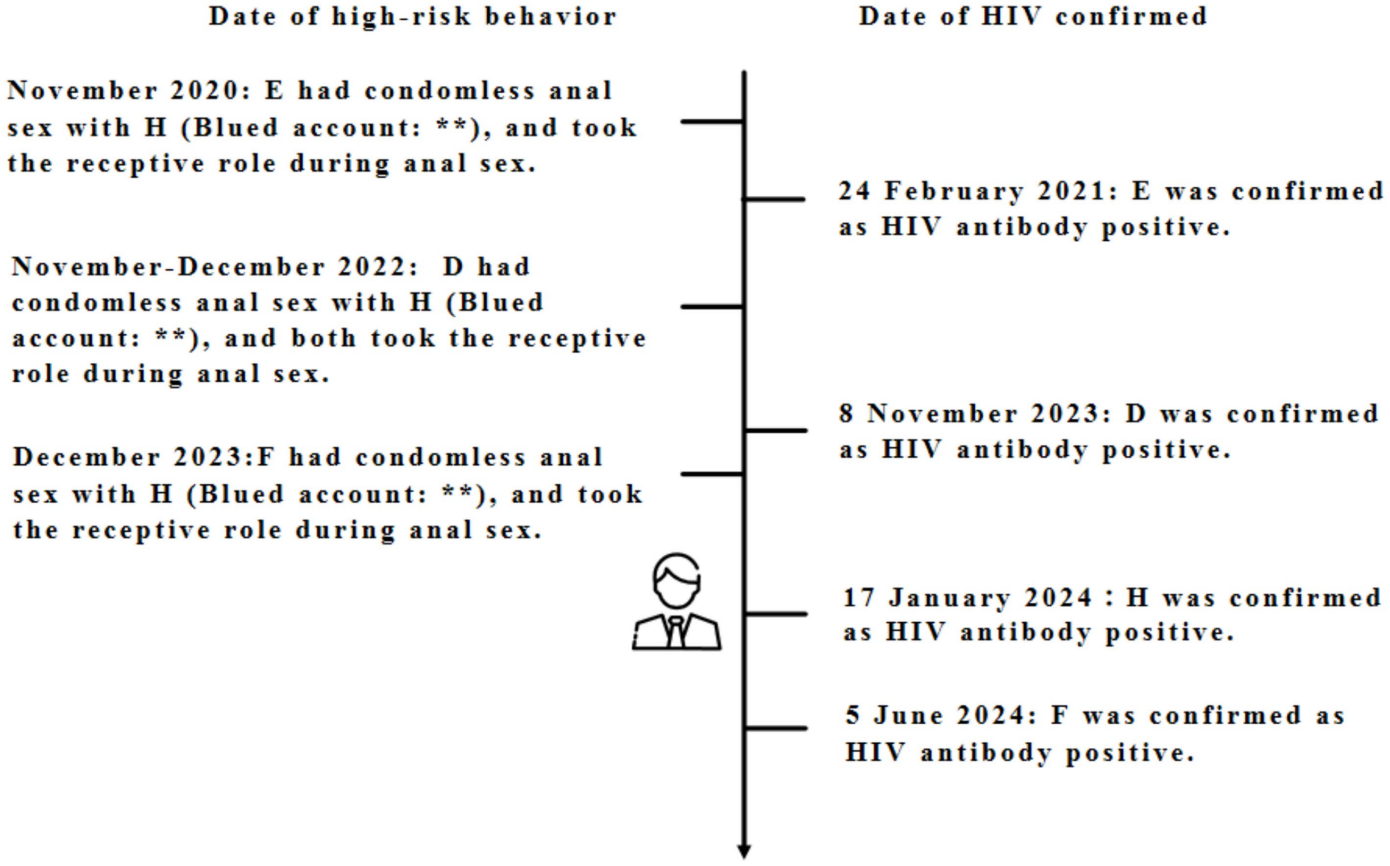
Timeline of high-risk sexual encounters and HIV diagnosis among **(D-F, H)**. The timeline is not to scale. This timeline depicts self-reported high-risk sexual encounters and HIV diagnosis dates; it does not confirm direct transmission between individuals, only potential epidemiological links.

After conducting sexual partner tracing (secondary follow-up) on H, he provided information on one high-risk sexual partner, who was identified as a previously confirmed local individual (D, a male young student, confirmed on November 8, 2023). A subsequent investigation by D revealed that he self-reported that H had actively solicited him for anal sex through Blued (in December 2022, with H taking the active role and removing the condom midway) (Figure 3).

By comparing the information of H with the high-risk sexual partner information collected in the local AIDS sexual partner tracing database from 2020 to 2024, it was found that H had epidemiological links with two other PLWH in the area (E, confirmed on February 24, 2021, who was invited by H through Blued and had unprotected anal sex in November 2020; F, confirmed on June 5, 2024, who was also invited by H through Blued and had unprotected anal sex in December 2023) (Figure 3).

After engaging in high-risk sexual behaviors without condom, none of the six young students used postexposure prophylaxis (PEP).

### 3.3 Molecular analysis of transmission links

Local molecular network surveillance protocols require follow-up institutions to collect pre-antiretroviral therapy (ART) plasma samples within 1 month of diagnosis. However, D, E, and F were residing outside the region at the time of diagnosis and follow-up, so pre-ART samples were not collected. Residual blood samples from their initial confirmatory tests (retained per national HIV testing guidelines) were used for sequencing after their identification via partner tracing.

**Figure 4:**
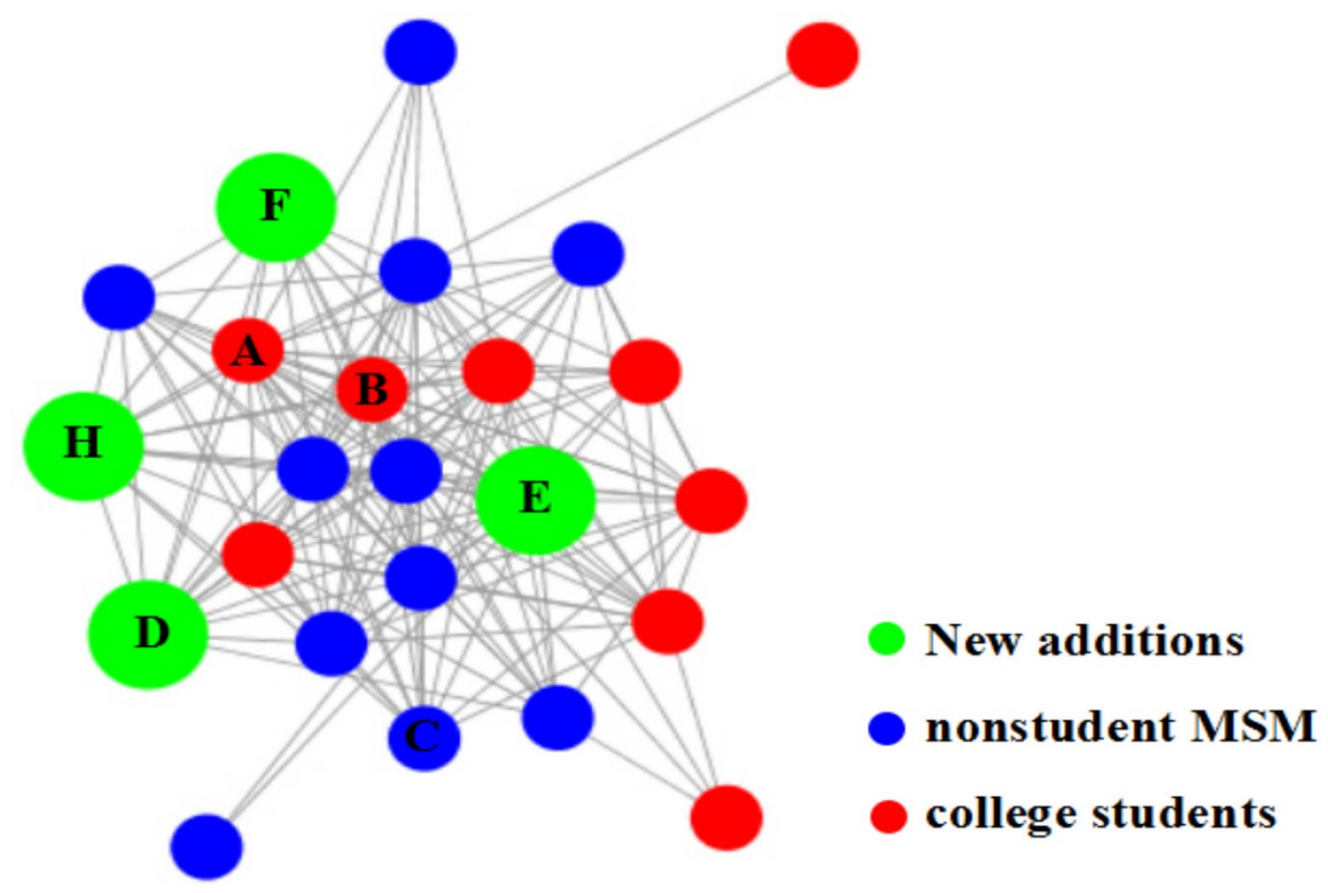
The key molecular cluster include new additions. Nodes represent the original 20 cluster members (red = young students; blue = nonstudent MSM) and new additions (H, D, E, F; orange = young students, green = H). Edges connect individuals with pairwise genetic distances ≤0.007 (consistent with Figure 1, the threshold for recent transmission). The inclusion of H, D, E, and F confirms their molecular relatedness to the original cluster at the predefined threshold for potential transmission links.

Laboratory molecular network analysis revealed that the samples from H and the aforementioned six young students belonged to the same molecular cluster (Figure 4).

**Figure 5:**
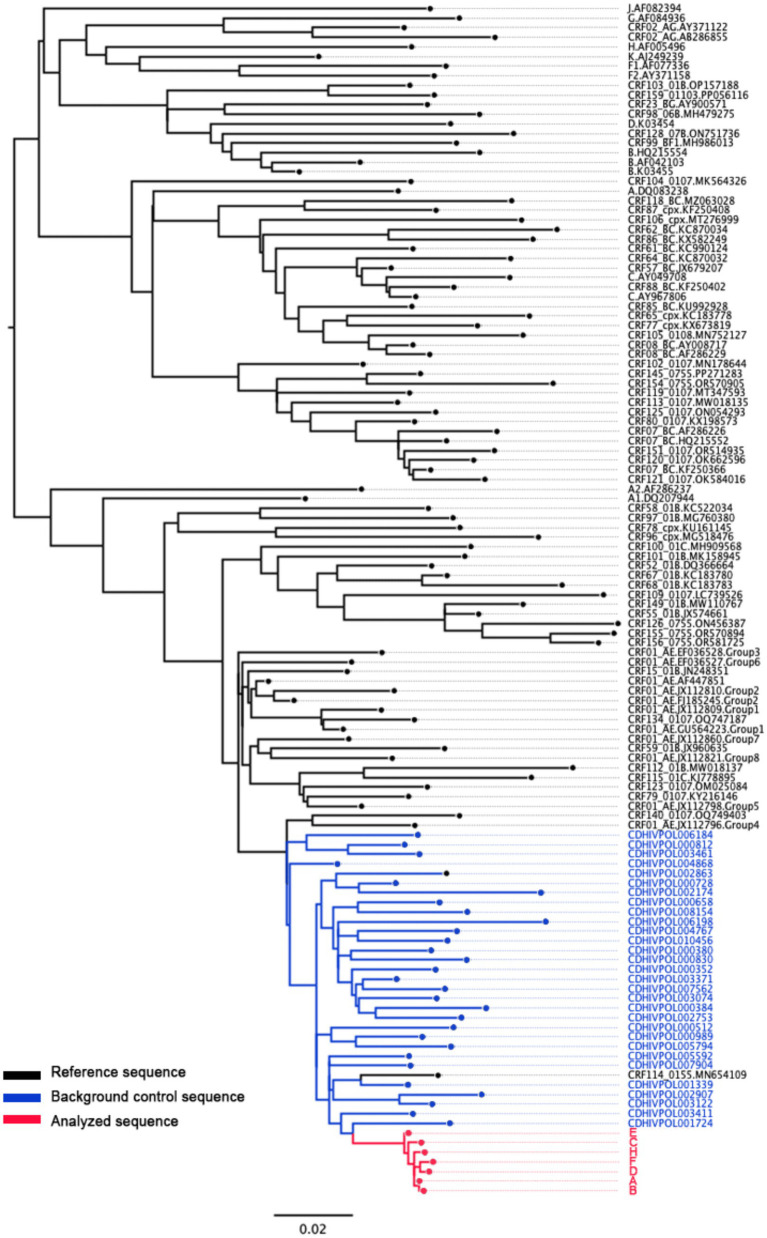
Phylogenetic tree of HIV-1 *pol* gene among H and 6 young students. Thirty background sequences (age 18–30 years, MSM transmission) with high genetic similarity and acceptable quality were selected from the laboratory database. Subtyping was performed using reference sequences of major HIV-1 subtypes obtained from the Los Alamos HIV Database.

The viral gene sequences from individual H and the six young students formed a distinct clade with a bootstrap support value of 95.6%. This clade is clearly separated from the background sequences used as controls, supporting that H and these 6 student cases form a closely related cluster, which is distinct from other MSM-associated HIV strains circulating in the same region during the same period. The genetic distances among these sequences were significantly smaller than those between them and the background control sequences (Figure 5). The results of the molecular network analysis and field epidemiology investigations were mutually corroborative.

## 4 Discussion

The central finding of our study underscores the value of integrating molecular transmission network analysis with in-depth epidemiological investigations to better understand transmission dynamics within specific populations. The case of Participant H illustrates how this integrated approach can reveal network connections that might remain undetected through molecular analysis alone. These findings suggest that combined methods may help identify individuals who could play an important role in transmission networks, enabling more timely and targeted public health interventions.

To our knowledge, studies combining detailed epidemiological investigations with comprehensive molecular analysis to better understand transmission dynamics within HIV clusters are scarce and are almost completely absent. Most studies have focused on the characteristics of molecular clusters to explore the regions and populations that need priority intervention (18–20). Our ability to identify and intervene promptly demonstrates the potential for precision public health interventions tailored to disrupt high-risk transmission networks. This approach has proven crucial in preventing further transmission, underscoring the importance of interdisciplinary strategies in HIV surveillance and control. Our research emphasizes the need for continued innovations in mapping and interrupting transmission pathways in real time.

Our findings reveal a concerning trend among young students and nonstudent MSM. In our study, the 5 young students diagnosed with HIV exhibited a limited perception of their risk for AIDS; these students engaged in unprotected anal receptive sex. None of these young individuals were on PrEP, and none sought out PEP following engagement in high risk sexual activity. The prevalence of unprotected anal sex and the lack of PEP utilization following such encounters highlight a profound lack of HIV prevention knowledge and risk perceptions among young students. This echoes findings from previous studies that have documented similar behaviors and low rates of condom use among young MSM, often fueled by social norms, peer pressure, and a misconception of low personal risk (21, 22). Consequently, our study underscores the urgency of implementing targeted interventions aimed at enhancing condom use, promoting awareness of PEP, and strengthening risk perception among young students.

In recent years, owing to the development of mobile phone and internet technology, more MSM are able to find sexual partners by using geosocial networking apps rather than via traditional methods (bathrooms, bars and other public places) (23–25). China’s sociocultural context has shaped unique risk scenarios. Blued is the first gay app originating from China and has more than 40 million registered users worldwide. It provides a quicker and easier way to find sexual partners who are geographically close but is also associated with high-risk sexual behaviors. Studies have shown that the use of geosocial networking apps is associated with high-risk sexual behaviors and HIV infection (26–29). Therefore, app-based interventions include information on HIV self-testing, preexposure prophylaxis (PrEP), PEP, and HIV prevention, which may benefit a broad group in the MSM community. The ubiquity of Blued and similar apps, while facilitating social connections for MSM, inadvertently fosters an environment conducive to risky sexual behaviors. We found that young students and nonstudent MSM were closely related through geosocial networking apps, which was in line with other studies (30). A previous study revealed a high prevalence (439/447, 98.2%) of geosocial networking app use among MSM attending university, which was much higher than that among nonstudent MSM (31). As a result, interventions should leverage the very platforms they utilize, integrating HIV prevention messages into the digital spaces where they congregate, thereby increasing the reach and impact of prevention efforts. Collaborations between health organizations and tech companies to develop user-friendly, culturally relevant, and evidence-based prevention interventions on Blued and similar apps hold great promise for reaching this high-risk group (32).

In addition, China has established a comprehensive HIV/AIDS response model involving a collaboration between the government, medical institutions, and community-based organizations—provides a solid institutional foundation for integrating molecular epidemiological surveillance with epidemiological investigations (14). After obtaining specific information on high-risk contacts, local MSM community organizations play a vital role in promoting HIV testing. These organizations act as one of the collaborators of the local public health system in delivering routine HIV testing, care, and follow-up services (33). When public health institutions identify newly diagnosed PLWH through routine testing, they can leverage the community networks and trust relationships of these organizations to assist in contacting relevant individuals within their social networks. Information exchange between researchers and community-based organizations adheres to the “minimum necessary” principle and is conducted under the supervision and ethical framework of public health institutions throughout the process, ensuring the protection of individuals’ privacy.

In conclusion, the present investigation provides several key insights with profound implications for HIV prevention strategies. First, the identification of hidden transmission hubs underscores the need for innovative surveillance methodologies that can swiftly detect and disrupt transmission networks. Second, the vulnerability of young students and nonstudent MSM highlights the importance of tailored interventions that address their unique needs and challenges, which emphasizes the importance of sexual partner tracing, as well as the timely identification of persons in need of HIV treatment and prevention services. Finally, the role of digital platforms in facilitating high-risk sexual behaviors necessitates a reevaluation of how we approach HIV prevention in the digital era, incorporating both technological solutions and social norm changes.

Some limitations to this study exist. First, there are five PLWH within the molecular cluster currently located in other regions who have yet to complete their epidemiological investigations, and the transmission chain may have been somewhat underestimated. Second, the last three young students diagnosed with HIV mentioned in the paper were not included in the initial cluster of 20 individuals for molecular network analysis due to the lack of blood samples collected prior to antiviral treatment, which serves as a reminder of the importance of timely blood sample collection to avoid any omissions. Third, the study was conducted in a specific geographical and demographic context, which may limit the generalizability of our findings. Future research should endeavor to replicate our methodology in diverse settings to assess its applicability.

## 5 Conclusion

By combining molecular transmission network analysis with epidemiological investigations, one individual with multiple high-risk sexual links who was initially not included in the molecular cluster was promptly detected, potentially preventing further transmission by creating awareness of HIV status and provision of HIV treatment services. It is necessary to improve the molecular network monitoring and conduct high-quality epidemiological investigation on the identified key molecular clusters as soon as possible to find persons who would benefit from HIV treatment and prevention services. Future research should assess the long-term impact of targeted interventions aimed at young students and nonstudent MSM, evaluating their effectiveness in reducing high-risk behaviors and ultimately curbing the spread of HIV.

## Data Availability

The original contributions presented in the study are included in the article/supplementary material, further inquiries can be directed to the corresponding author.
